# Facial Prominence of Political Candidates: Gender Differences in Private and Public Pages on Facebook Profile

**DOI:** 10.3389/fpsyg.2021.737916

**Published:** 2021-10-18

**Authors:** Alessia Valmori, Tania Garau, Luciana Carraro, Luigi Castelli

**Affiliations:** Department of Social and Developmental Psychology, University of Padova, Padova, Italy

**Keywords:** politics, non-verbal, social networks, gender differences, face-ism

## Abstract

The face of a person is an important source of communication and information especially for politicians who are continuously portrayed through media. Voters may use this information to form an impression about the candidates and several inferences may be drawn. Within this frame, research has largely investigated gender differences. One line of research has focused on the facial prominence of people portrayed in pictures, describing a tendency to portray men with a higher face prominence as compared to women. This bias has been defined as the face-ism effect and it has a key influence on the perception of dominance, competence, intelligence, and ambition of the portrayed individuals. Several studies in recent years analyzed the differences between the self-representation of men and women in social media, but no study specifically focused on politicians directly comparing two different types of profiles: private vs. public. In two studies, we analyzed differences in face-ism index comparing male and female politicians both for pictures posted in private and public Facebook accounts. In Study 1 results showed that no difference emerged between men and women engaged in politics when considering public pages; however, when private profiles are analyzed, women displayed a higher face-ism index than their male counterparts. Study 2 partially confirmed results from Study 1 considering Italian politicians. Overall, current results showed a different pattern as compared to previous studies suggesting an increase in perceived agency and dominance for female candidates, particularly emphasized in their private profiles.

## Introduction

Face-ism is a largely investigated concept in social psychology and it is related to the facial prominence of individuals portrayed in paintings, pictures, or other visual media. More specifically, face-ism is operationalized as the ratio between the space occupied by the face and the space devoted to the representation of the body (Archer et al., 1983). The key interest for face-ism lies in the empirical demonstration that the representations of members of minority groups are often associated to lower facial prominence (e.g., Zuckerman and Kieffer, 1994). Most studies have investigated gender-based differences and, across time and cultures, it has been shown that men tend to be represented with higher facial prominence as compared to women (e.g., Archer et al., 1983; Zuckerman, 1986; Nigro et al., 1988; Dodd et al., 1989). Importantly, there is evidence from anthropometric data that these findings cannot be explained by any eventual gender difference in actual body proportions (Konrath and Schwarz, 2007). For this reason, face-ism has been considered as a subtle form of gender stereotyping. Indeed, there is evidence that a high degree of facial prominence is associated with a perception of greater competence, intelligence, assertiveness, ambition, and dominance (Archer et al., 1983; Zuckerman, 1986; Schwarz and Kurz, 1989), whereas the focus on the body strengthens the sexualization and objectification of women (Fredrickson and Roberts, 1997). Therefore, face-ism has been considered as a reflection of pervasive gender stereotypes but also as a subtle way in which they are continuously reinforced through the exposure to media that differently portray men and women. As a consequence, women might find it more difficult to succeed in domains in which stereotypically masculine traits, such as competence and dominance, are maximally valued. For instance, it is well-established that inferences of competence and dominance from the physical aspect of a candidate can strongly influence voting decisions and the choice of one’s own leader (e.g., Todorov et al., 2005; Castelli et al., 2009; Antonakis and Eubanks, 2017); thus, female and male candidates in political races may elicit different impressions among the electorate if portrayed in a biased way in terms of facial prominence. In most countries, male politicians are indeed represented with higher facial prominence as compared to female politicians (Adams et al., 1980; Konrath and Schwarz, 2007; Szillis and Stahlberg, 2007; Konrath et al., 2012).

This biased representation may represent an additional obstacle for women in their access to political offices, and therefore, it becomes crucial to identify and, possibly counteract, any bias that could impede equal access to political careers and reinforce gender gaps in political officeholding. The underrepresentation of women in politics is a major social issue in most countries at both the national (e.g., Jalalzai, 2013; UN Women Headquarters, 2020) and local level (e.g., Ceciarini, 2019). In the present work, we focused on municipal elections as a first key step of involvement at the community level. In particular, we focused on two distinct European countries – Italy and Finland – that are characterized by a different history and current situations as for the participation of women in the political life. For instance, Finnish women and men gained full political rights in 1906, whereas in Italy this happened only after the II World War. Similarly, whereas Finland has already been led by three women Prime Ministers, only male politicians have so far led Italian governments. At the local/municipal level, 39% of the leaders are women in Finland, whereas the percentage drops to 15.3% in Italy (EIGE, 2021), despite the presence in Italy of gender quotas in the formation of the party lists (Law 215/2012). These differences are also reflected in the Gender Equality index (EIGE, 2020) showing that Finland scores higher (74.7) compared to the EU mean (67.9) and, more importantly, compared to Italy (63.5). The gap between the two countries is even more evident when considering political power (FI: 83.9; IT: 49.3), even though no relevant differences emerge as for the level of education (FI: 61.6; IT: 61.9). Hence, the analysis of these different national contexts in terms of women participation to the political life can allow to explore the generalizability of the effects under scrutiny.

### Face-ism in Social Networks

In recent years, the analysis of the face-ism effect has been extended to social networks that offer the opportunity to perform analyses on massive databases of photographs. Most importantly, on social network sites users typically can actively choose the photographs to publish and how to eventually crop them. Pictures appearing on newspapers or institution websites are in most cases likely to reflect the choices and stereotypes of photographers, editors, or website managers who decide the images to be published and how to frame them. In sum, they are more closely tied to social stereotypes about gender roles. In contrast, the analysis of pictures posted in social networks should more specifically reflect the users’ intentions and their self-presentation strategies, namely how they want to be perceived by external observers. In an extensive study, Smith and Cooley (2012) analyzed the face-ism index of men and women on different online social networking sites from seven countries showing that men pervasively tended to portray themselves with greater face-ism index compared to women (see also Bakan and Bakan, 2018). Čuš Babič et al. (2018) focused on selfies posted on Instagram and found a similar gender bias. This is in line with research findings indicating that women’s selfies do often present forms of self-objectification and conformity to gender stereotypes, with a frequent exposure of body parts and the face obscured (Döring et al., 2016; Bell et al., 2018; Butkowski et al., 2020; see also Cooley and Smith, 2013).

Overall, the available research suggests that, despite the possibility for social network users to self-select the way they want to present themselves to others, women continue to often self-objectify and to display a reduced facial prominence as compared to men. This strongly points to an internalization of gender stereotypes and to what has been called “self-inflated face-ism” (Smith and Cooley, 2012). This effect emerged also in a controlled laboratory study in which male and female participants were allowed to digitally modify their photographs to be allegedly posted in an online professional network (Sczesny and Kaufmann, 2018).

The analysis of self-representations within the political domain is particularly interesting for several reasons. Indeed, women who decide to run for political offices may be characterized by specific motivational and personality profiles. For instance, Conroy and Green (2020) showed that women candidates employ more agentic words compared to women noncandidates. In addition, they found that differences in agency and communion between candidates and non-candidates were greater compared to those between women and men, supporting the idea that female politicians who run for political offices are characterized by overall high levels of agency. In a related vein, Coffé and Bolzendahl (2021) showed that women who score high in agentic traits also display more active political engagement thus highlighting again the strict link between agency (vs. communality) and political participation.

This would lead to predict that the differences in facial prominence between male and female candidates could disappear, or even reverse, when female politicians can actively choose the visual representation to be included in social media. In line with this hypothesis, Jungblut and Haim (2021) have recently analyzed the Twitter and Instagram accounts of candidates running in the 2019 European Election showing no significant difference between male and female candidates in terms of facial dominance (i.e., whether the face occupied at least one third of the picture). Similarly, Brands et al. (2021) found that the pictures of Dutch and American politicians posted in Instagram did not differ as a function of gender in relation to camera perspective (i.e., close-ups vs. full body shots).

With few exceptions (e.g., Brands et al., 2021; Jungblut and Haim, 2021), previous research about face-ism has mainly considered the facial prominence of male and female politicians in institutional websites (e.g., official government websites). In the present work, we will further investigate how politicians are portrayed in a popular social network, namely Facebook. Facebook is the most popular and employed social network both in Finland and in Italy (StatCounter Global Stats, 2018) and, according to Shepherd (2016), women are more prone to reflect on their Facebook activity as compared to men. Critically, Facebook is also a unique social media platform because it allows to create two different types of accounts: Private and public profiles. The private profile allows users to control who can connect with their profile (i.e., “become friends”) and therefore have access to the contents shared on the private wall. In contrast, the public page is an open profile and therefore all the contents are public. In addition, whereas the contents on the personal profile reflect the unconstrained choices of the owner, the public profiles of the politicians are more likely influenced by the interventions of staff members, and therefore these profiles may also reflect the gender stereotypes of third parties. Although several studies have been dealing with the face-ism effect in social media, to the best of our knowledge, no previous study has analyzed politicians’ social media profiles as a function of whether the profile is personal in nature, or it is related to the public activity of the person as a politician. Overall, in line with Brands et al. (2021) and Jungblut and Haim (2021), we expected to observe no higher facial dominance of male as compared to female candidates. More specifically, although we had no *a priori* hypotheses, we also explored whether face-ism in the more informal private profiles. Two competing hypotheses could be put forward. On the one hand, female candidates may self-present with the same level of facial prominence as male candidates only in the public profile, namely when they use it as a tool within the political campaign and are thus motivated to appear as agentic, whereas the typical face-ism effect still appears in the private setting, as often shown in the case non-politicians (e.g., Smith and Cooley, 2012; Čuš Babič et al., 2018). On the other hand, observing that female candidates counteract visual stereotypes also in their private profiles would provide support to the idea that even in unconstrained settings, female politicians act as highly agentic individuals, overcoming the aforementioned “self-inflated face-ism” (Smith and Cooley, 2012). Importantly, the presence in Facebook of two distinct social media profiles allows not only to analyze whether the overall level of gender differences in terms of facial prominence varies as a function of the nature of the setting (public vs. private), but it also provides the opportunity to assess whether self-presentation formats tend to be consistent across profiles. In other words, it enables to explore the correlation between the facial prominences displayed in the two profiles and, therefore, whether relatively stable individual differences can be detected in the way close-ups are selected when self-presenting to others.

### The Role of Age in the Face-ism Effect

Previous work has shown that the face-ism index may change as a function of the age of the persons portrayed (Szillis and Stahlberg, 2007; Prieler and Kohlbacher, 2017; Read et al., 2017). Some studies found that older women tend to have a higher facial prominence than younger women (Szillis and Stahlberg, 2007; Read et al., 2017) whereas men are depicted with the same facial prominence irrespective of their age (Szillis and Stahlberg, 2007). In another study, instead, it emerged that facial prominence tends to decline for women with age (Prieler and Kohlbacher, 2017). Although previous research findings do not allow to make clear hypotheses, we took an exploratory approach to test whether age can indeed modulate the facial prominence of male and female politicians.

## Study 1

### Method

**Figure 1:**
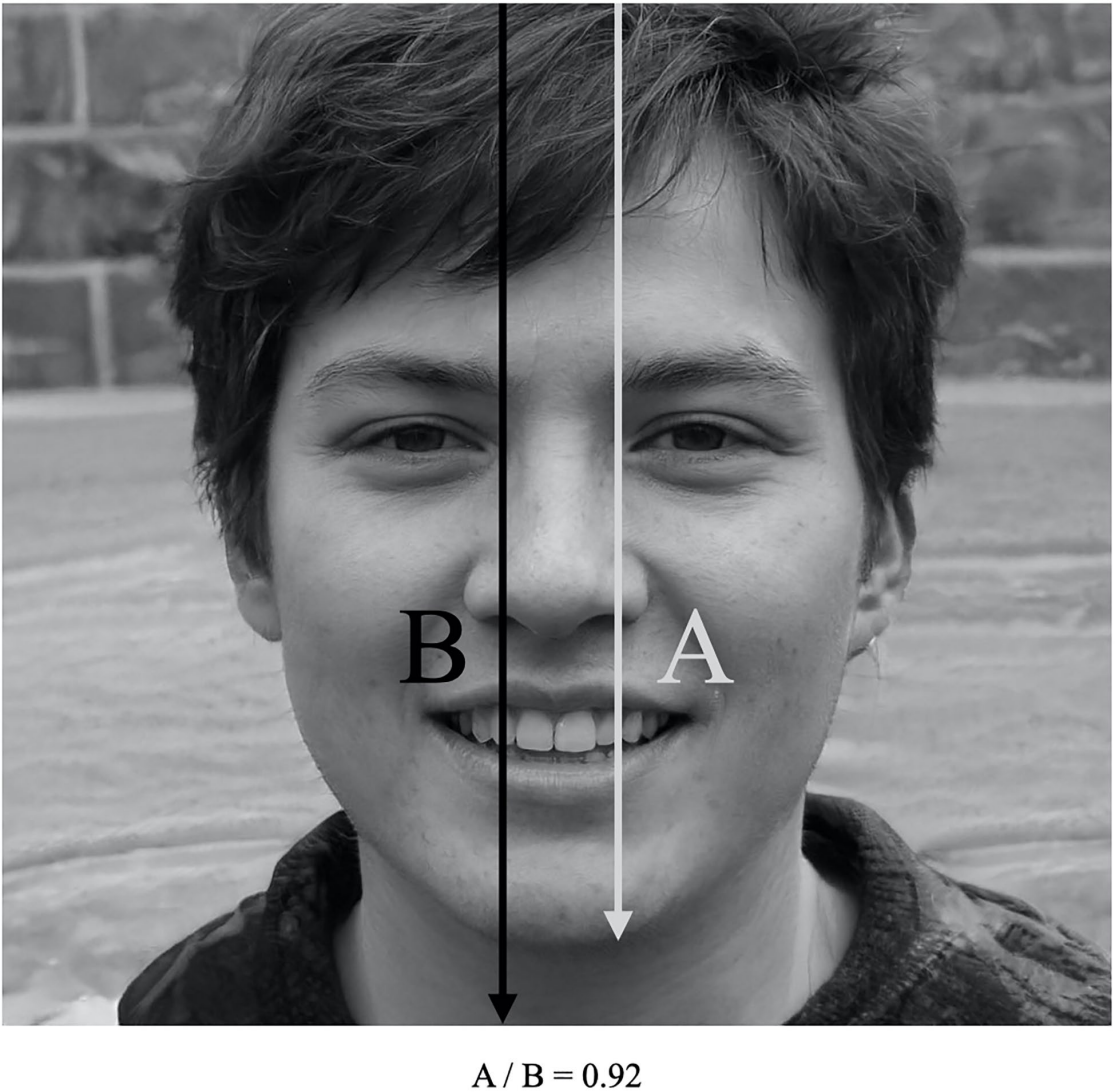
Example of the two indexes calculated for each profile picture in order to compute the face-ism index.

We considered a sample of 9,560 candidates (*N*_Male_=5,379, *N*_Female_=4,181) for the Finland municipal election run in 2017 (for details see Table 1). The face-ism index was calculated by two independent judges using the Page Ruler extension for Google Chrome that allows to obtain accurate and objective measures based on pixels. In order to test reliability, both judges calculated the face-ism index on a subset of photographs (*N*=50, *α*=0.93). We measured the space in the picture occupied by the face (called “A,” i.e., the distance from the top of the head to the lowest point of the chin) and the space occupied by the whole body of the person portrayed (called “B,” i.e., the distance from the top of the head to the lowest visible part of the body; see Figure 1). The face-ism index was operationalized as the ratio between A and B; therefore, higher values indicate a greater facial prominence.

**Table 1:** Frequencies for private profiles and public pages together with means and SDs for age, friends, and followers divided for country and gender.

	Age	Private profile (with pictures of themselves)	Public page (with pictures of themselves)	Both private profile and public page (with pictures of themselves)	Friends in private profile	Followers in public page
Finland	*M* =44.59 (*SD* =12.96)	6,216 (4,921)	2,662 (2,343)	2,009 (1,500)	*M* =705.19 (*SD* =804.25)	*M* =512.45 (*SD* =2,624.59)
Men	*M* =45.17 (*SD* =13.37)	3,418 (2,710)	1,319 (1,143)	988 (742)	*M* =723.55 (*SD* =832.23)	*M* =545.73 (*SD* =3,038.08)
Women	*M* =43.84 (*SD* =12.38)	2,798 (2,211)	1,343 (1,200)	1,021 (758)	*M* =677.67 (*SD* =759.94)	*M* =484.88 (*SD* =2,226.00)
Italy	*M* =52.77 (*SD* =10.74)	5,226 (3,406)	2,223 (945)	1,695 (651)	*M* =1,971.36 (*SD* =1,655.23)	*M* =4,057.77 (*SD* =29,852.63)
Men	*M* =53.03 (*SD* =10.86)	4,285 (2,902)	1,820 (819)	1,383 (562)	*M* =2,011.03 (*SD* =1,672.92)	*M* =3,354.66 (*SD* =14,397.21)
Women	*M* =51.12 (*SD* =9.79)	668 (504)	291 (126)	225 (89)	*M* =1,745.71 (*SD* =1,533.18)	*M* =8,434.66 (*SD* =71,792.49)

### Results

Because not all candidates had both a private and a public profile, two different approaches have been followed in the analyses. First, we separately analyzed the face-ism index for the public and private profile pictures. A significant gender difference emerged in the private profiles, *t*(4,919)=−5.089, *p*<0.001, *d*=0.147. Women displayed a higher face-ism index (*M*=0.627, *SD*=0.223) as compared to men (*M*=0.593, *SD*=0.236). When considering the public profiles no significant difference between men (*M*=0.630, *SD*=0.195) and women (*M*=0.638, *SD*=0.186) emerged, *t*(2,340)=−0.931, *p*=0.352, *d*=0.038.

**Figure 2:**
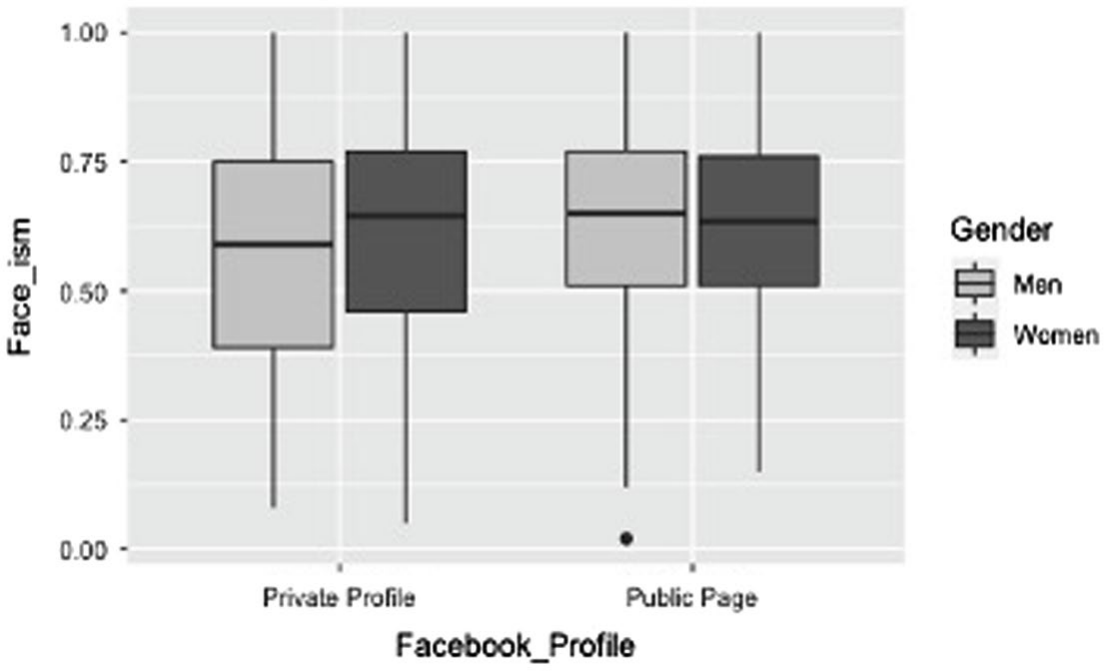
Two-way interaction between gender and type of Facebook profile on face-ism index emerged in Study 1.

Next, we focused on candidates who used both types of Facebook. Face-ism indexes were submitted to a 2 (gender: men vs. women)×2 (type of profile: private vs. public) ANOVA with the latter factor varying within participants. A significant main effect of gender emerged, *F*(1,1,499)=5.66, *p*=0.017, *η^2^_p_* =0.004. Women generally presented a higher face-ism index (*M*=0.622, *SD*=0.165) as compared to men (*M*=0.602, *SD*=0.164). A main effect of the type of profile also emerged, *F*(1,1,499)=22.34, *p*<0.001, *η^2^_p_* =0.015, due to higher face-ism indexes in the public (*M*=0.628, *SD*=0.194) than private profiles (*M*=0.596, *SD*=0.232). Importantly, the analysis also yielded a significant two-way interaction between gender and the type of the profile, *F*(1,1,499)=8.90, *p*=0.003, *η^2^_p_* =0.006 (see Figure 2). As for private profiles, men presented lower face-ism indexes (*M*=0.576, *SD*=0.218) as compared to women (*M*=0.616, *SD*=0.220), Bonferroni *post-hoc* comparisons *p*<0.001. No significant difference emerged when considering public pages (*p*=0.971). Likewise, men registered lower face-ism index when their profile was private (*M*=0.576, *SD*=0.218) rather than public (*M*=0.627, *SD*=0.191; Bonferroni *post-hoc* comparisons *p*<0.001), whereas no significant difference emerged when considering the public and private profiles of women (*p*=0.216; see Figure 2).

In order to examine the link between the face-ism index in the public and private profiles, we first excluded all the cases in which the same picture appeared in both profiles. The correlation was significant when considering the whole sample, *r*(1,378)=0.201, *p*<0.001, and it had the very same magnitude for both male and female politicians (*r*s=0.201).

#### The Role of Candidate’s Age

There was no significant difference in the mean age of men and women who used Facebook (*M*=43.80 and 43.21, respectively). Age was significantly and positively correlated to face-ism indexes for both male and female candidates. More specifically, as for the private pages, the correlation was 0.140 and 0.142, for male and female candidates, respectively (*ps*<0.001); for the public pages, the correlation was 0.089 and 0.178, for male and female candidates, respectively (*ps*<0.005). Next, the same 2 (gender)×2 (type of profile) ANOVA described above was carried out on the face-ism index including age as a covariate. The results remained unchanged. Indeed, the key two-way interaction between gender and profile was still significant, *F*(1,1,498)=8.672, *p*=0.003, *η^2^_p_* =0.006, despite a strong main effect of age, *F*(1,1,498)=63.25, *p*<0.001, *η^2^_p_* =0.041. Hence, although there was an overall trend of increasing facial prominence with age, the previously described pattern of findings was still observable. The correlation between the face-ism index in the two profiles remained significant after controlling for age, *r*(1375)=0.182, *p*<0.001.

### Discussion

In this study, we focused on the self-presentation of political candidates on Facebook analyzing gender differences in facial prominence in their profile pictures. Overall, no reliable gender differences emerged in their public profiles, whereas the analysis of the private profiles showed that female candidates presented themselves with a higher facial prominence as compared to male candidates. These results can be interpreted as a signal that women engaged in politics may actually overcome gender stereotypes that could impact on the head–body ratio while self-presenting on social networks especially in their private profile pages.

In a follow-up study, we aimed to further explore the consistency of these findings in a different social context, namely a country (i.e., Italy) in which women are still heavily underrepresented in political life.

## Study 2

### Method

The list of the 7,998 Italian mayors (*N*_Male_=6,519, *N*_Female_=1,033) was retrieved in April 2018 from the following website: http://www.comuni-italiani.it/amm/sindaci.html. For some cities (i.e., 446), the position was vacant because while in Finland the elections occur once every 5years for all the municipalities, municipal elections in Italy are held at different times. For each mayor, we considered both the private and the public Facebook profiles (see Table 1). The face-ism index was calculated by four independent judges following the same procedure adopted in Study 1. Two judges calculated the face-ism index on a same subset of photographs showing a high degree of concordance (*N*=50, *α*=0.99).

### Results and Discussion

First, we separately analyzed the face-ism index in the private and public Facebook profiles. As for the private profiles, females displayed a higher face-ism index (*M*=0.537, *SD*=0.226) as compared to males (*M*=0.510, *SD*=0.224), *t*(3,404)=− 2.48, *p*=0.013, *d*=0.119. In contrast, as for the public page, no significant difference between male (*M*=0.521, *SD*=0.192) and female (*M*=0.509, *SD*=0.187) politicians emerged, *t*(943)=0.65, *p*=0.518, *d*=0.063. As in Study 1, we selected the cases in which different images were displayed in the public and private profiles. The correlation between the two indexes was significant when considering the whole sample, *r*(578)=0.240, *p*<0.001, and it had a rather similar magnitude for male and female politicians (*r*=0.248 and 0.178, respectively).

Next, we examined the data about the mayors who had included their pictures in both types of profiles. The face-ism index was submitted to a 2 (gender: men vs. women)×2 (type of profile: private vs. public) ANOVA with the latter factor varying within participants. No significant results emerged (all *p*s>0.44). Moreover, in no case, the face-ism indexes correlated with the politicians’ age (all *p*s>0.17), and the correlation between the face-ism indexes in the two profiles remained significant after controlling for age, *r*(575)=0.240, *p*<0.001.

Overall, Study 2 revealed a tendency for women to show a greater facial prominence in their private profiles compared to men, but no differences emerged when considering the public pages. The pattern was similar to the one observed in Study 1, although findings were far less definite, and they should therefore be treated more cautiously. This was likely due to a general reduced presence of female politicians and, as a consequence, the number of female mayors who had both public and private accounts was rather limited.

## General Discussion

The present findings contribute to enriching the literature about gender differences in the visual self-presentation of political figures in social networks. Previous studies have explored the face-ism effect in online images in relation to the general population, showing a pervasive higher facial prominence in the case of men (e.g., Smith and Cooley, 2012; Čuš Babič et al., 2018). However, political candidates often present specific characteristics as compared to the general population (e.g., Caprara et al., 2003). In line with this view, Conroy and Green (2020) have stressed how female candidates might be more agentic than women with lower political ambition. It can thus be important to focus on how politicians present themselves in social media, analyzing also the phenomenon in different contexts that are characterized by variable levels of personal control and motivation over the transmitted contents. Facebook represents a unique social network in this regard in that it allows to convey information through two different types of profile: One is strictly personal whereas the other is public and can reasonably reflect the interaction between the politician and the staff members in order to effectively shape the political campaign. As for the public profiles, results from both studies indicated that there was no effect of gender on facial prominence, confirming the findings obtained by Jungblut and Haim (2021) in relation to European Elections (see also Brands et al., 2021). Hence, there was no evidence of any gender-based discrepancy in the way male and female politicians visually present themselves. Critically, this was true in both the countries that have been considered here, despite their strong differences in relation to the level of active participation of men and women in the political life. When there is some level of proximal control over the choice of the personal image to display to the followers, gender differences in terms of facial prominence seem to disappear. Differences, however, may still be present in websites that are managed by external sources such as institutional websites. For instance, an examination of the pictures of the 200 politicians in the website of the Finnish Parliament, and of a random sample of 200 politicians in the website of the Italian Parliament (*N*_FinMen_=109, *N*_FinWomen_=91; *N*_ItaMen_=119, *N*_ItaWomen_=81), revealed that men (*M*=0.654, *SD*=0.003) are represented with a marked higher facial prominence as compared to women (*M*=0.611, *SD*=0.004), *F*(1,396)=66.158, *p*<0.001, *η^2^_p_* =0.143. The interaction between gender and country was not significant (*p*=0.54, *η^2^_p_* =0.001), indicating a pervasive face-ism in both countries. In sum, it appears that male and female politicians continue to be differentially portrayed in institutional websites in accordance with cultural stereotypes. However, in social networks that enable to make choices about how to visually present themselves, male politicians no longer display a higher facial prominence. This has important implications because facial prominence may have a significant impact on the perception of key personality characteristics of the person portrayed, such as competence and agency (e.g., Schwarz and Kurz, 1989), which, in turn, may affect the chances of electoral success. Whereas an association between female politicians and typically masculine issues and domains can be detrimental and trigger backlash against women (Okimoto and Brescoll, 2010; Bauer and Carpinella, 2018), strategies aimed at de-emphasizing the possession of feminine traits can enhance women’s likelihood of winning elected offices (Bauer, 2020).

In an exploratory way, we also examined the private profiles of the politicians. This analysis is highly informative for several reasons. First, the images posted in the private profiles are probably more likely to reflect personal choices that are not influenced by third parties. Second, private profiles allow to assess the self-presentation choices of men and women engaged in politics when they do not directly speak to an audience as political figures. Findings in both countries showed that, on private profiles, women even achieved higher levels of facial prominence as compared to men. This suggests that in informal contexts which are less likely influenced by strategic choices aimed at attracting electoral consensus, women who actively participate in the political life do actually visually self-present as agentic individuals. Previous research on political elites has shown that female politicians score higher than the general population on the energy/extraversion factor of the Big Five indicating their high levels of activity, competitiveness, and vigor (Caprara et al., 2010; see also Conroy and Green, 2020). It can thus be tentatively concluded that women who overcome the barriers that obstacle their access to politics present particularly high levels of agency and this is reflected in the unconstrained choices they make when selecting or cropping the personal image to be displayed in their personal profiles. The fact that the face-ism indexes in the two profiles were significantly correlated is consistent with the idea that individual differences do pervasively shape the choices about the pictures to display in social media.

Some limitations of the present studies, however, should be acknowledged. First, the sample of Italian mayors was not sufficiently balanced in terms of gender composition and therefore the limited number of observations in relation to female mayor invites caution in the interpretation of the findings. Second, no information was available about who was actually responsible for the pictures posted in the public profiles, thus making impossible to determine the respective role of the politicians and their staff. Next, our analysis focused only on two European countries, in order to generalize our results, it would be important to extend the analysis to other countries. Finally, we had no direct information about the personality characteristics of the politicians. Future studies will benefit from a more specific analysis of how the personality features of politicians as well their motivations (see Wu et al., 2015) may affect their visual self-presentation in social media.

## Conclusion

Political communication has largely changed in the last few decades with an increasing use of social media to self-promote and remain in contact with the electorate, as well to provide contents for the other media that cover the political debates (e.g., Dimitrova and Matthes, 2018). In the present work, we have focused on a specific facet related to the transmission of visual information through Facebook, and, more specifically, on facial prominence. No gender difference was observable on public profiles, and the typical face-ism effect was reversed in the private profiles. With respect to the specific domain of facial prominence, it thus appears that women involved in politics self-present in a way that do not undermine their perceived agency.

## Data Availability Statement

The raw data supporting the conclusions of this article will be made available by the authors, without undue reservation.

## Ethics Statement

Ethical review and approval was not required for the study on human participants in accordance with the local legislation and institutional requirements. Written informed consent was not required to participate in this study in accordance with the national legislation and the institutional requirements.

## Author Contributions

AV, TG, LCr, and LCs conceived the studies, performed the analysis, and wrote the manuscript. All authors contributed to the article and approved the submitted version.

## Funding

This study was supported by the Visiting Programme of the Cariparo foundation number CUP C96C18004880005.

## Conflict of Interest

The authors declare that the research was conducted in the absence of any commercial or financial relationships that could be construed as a potential conflict of interest.

## Publisher’s Note

All claims expressed in this article are solely those of the authors and do not necessarily represent those of their affiliated organizations, or those of the publisher, the editors and the reviewers. Any product that may be evaluated in this article, or claim that may be made by its manufacturer, is not guaranteed or endorsed by the publisher.
